# Factors Associated with Medication Noncompliance in Dogs in New Zealand

**DOI:** 10.3390/ani14172557

**Published:** 2024-09-03

**Authors:** Thomas F. Odom, Christopher B. Riley, Jackie Benschop, Kate E. Hill

**Affiliations:** 1 School of Veterinary Science, Massey University, Palmerston North 4442, New Zealand; criley03@uoguelph.ca (C.B.R.);; 2 Ontario Veterinary College, University of Guelph, Guelph, ON N1G 2W1, Canada

**Keywords:** compliance, adherence, persistence, dog, pet owner, medication, survey

## Abstract

**Simple Summary:**

Factors affecting dog owners’ compliance with veterinary medication recommendations were examined, and barriers and aids to administering medications were identified. Findings revealed that older dogs had more compliant owners, and clients who used no aids for medication were more likely to be compliant. Almost half of owners reported not being shown how to administer the medication, and one-third faced challenges, mainly due to resistant pets. Nearly half of the surveyed clients were noncompliant, which is higher than previously reported in other studies. Our study highlights the necessity of enhancing client compliance with veterinary medication instructions for dogs. It emphasizes the importance of providing standardized instructions and guidance, including demonstrating how to administer medications. Ensuring that prescribed oral medications are palatable and easy to administer before patient discharge may contribute to improved patient outcomes.

**Abstract:**

Client compliance with prescribed medication instructions to treat their pets is a concern. This study describes factors associated with the noncompliance of dog owners with veterinary recommendations for medication, as well as client-reported barriers and aids to administering medications. A cross-sectional survey of dog owners’ compliance with veterinary medication recommendations was performed from 9 January 2019 to 18 July 2020. A convenience sample of owners who prescribed medication for their dogs during or following elective veterinary examination was surveyed regarding medication administration experience and compliance. Owners were followed up to determine if the course of medication had been completed. Compliance data were analyzed descriptively. Logistic regression was performed with compliance as the outcome. Medication noncompliance was recorded for 47% (71/151) of owners. Increasing dog age was associated with better owner compliance (*p* < 0.05). Pet owners who used “nothing” as an aid to medicating were less likely to be noncompliant (*p* < 0.05). Forty-seven percent (71/151) of owners reported that “nobody” showed them how to administer the medication. One-third of dog owners (47/151) reported challenges in medicating their pets. The most common reason cited by clients reporting challenges was a resistant pet. Demonstration of medication administration techniques and discussion about available aids to medicating a pet may improve client compliance.

## 1. Introduction

Medication administration by clients is an essential aspect of pet healthcare, and it is crucial that pet owners follow the instructions of their veterinarian for the optimal response to treatment [1,2]. However, compliance with veterinary directions for medication administration to dogs has been an issue of concern for many years, as it is often challenging for pet owners to ensure that their pets receive the correct dosage and adhere to the medication schedule [3].

There are few published studies in veterinary medicine where potential factors affecting compliance have been tested. Those published have suggested that compliance is improved when the pet owners feel the veterinarian has spent enough time with them in the consultation, when information sheets are delivered with the prescription, and when important information and the explanation of the effects of the medication is repeated during the consultation [4,5,6]. Several studies have suggested that the complexity of the medication regimen has an impact on compliance [5,7,8]. For example, one study of 51 owners found high compliance with correct dosing (>90%) but low compliance with the prescribed timing of medication administration (22%) [5]. Another reported frequency of medication affected compliance, with 100% compliance in a sample of 90 clients, found nine times more likely for antimicrobials required to be given one to two times daily compared with three times a day [7]. However, a recent systematic review of factors affecting owner compliance with medication treatment recommendations found that evidence available regarding factors affecting client compliance with pharmaceutical treatment recommendations in dogs and cats is limited and of poor quality [3]. Few studies have investigated owner-related human factors associated with poor compliance [9,10] or the role of the human–animal bond and medication administration [11,12]. Noncompliance with medication regimes may impact the health and well-being of the pet and lead to increased treatment costs for the client [2,13,14]. Improved pet owner compliance has the potential to improve clinical and welfare outcomes for pets, facilitate timely preventive medicine, and ensure a better quality of life for pet dogs and owners [7].

The aims of this study were to identify factors associated with the noncompliance of owners with medication administration instructions for their dogs, to evaluate associated factors, and to provide context to client-reported barriers and aids to the administration of medications in a New Zealand primary care practice. Based on the findings, the authors seek to provide insights into the importance of medication compliance in dogs and offer recommendations for veterinarians and pet owners to ensure that their pets receive the best possible care.

## 2. Materials and Methods

### 2.1. Study and Survey Design

A prospective cross-sectional study of the compliance of dog owners with veterinary medication recommendations was performed from 9 January 2019 to 18 July 2020, using a convenience sample of clients who were prescribed medication for their pets by veterinarians of the Massey University Veterinary Teaching Hospital (MUVTH) during or following an elective veterinary examination. An online questionnaire was developed using proprietary software (Version 10/2018) (Qualtrics XM, Seattle, WA, USA; www.qualtrics.com) that encompassed owner demographics, queries relating to the administration of medications prescribed for their dog, and identification of challenges associated with complying with veterinary directions (Supplementary File S1). Questions were provided with binary, categorical, or semiquantitative response options. Those relating to medication compliance used Likert scales, while questions relating to challenges and barriers allowed for multiple selections and free-text entry. Five staff members employed at MUVTH were invited to pilot the questionnaire, and the feedback was used to refine questions in an iterative process to ensure valid questions.

### 2.2. Inclusion and Exclusion Criteria

Clients were invited to participate in this study if their dog was seen by the MUVTH Community Practice veterinarian and had been prescribed oral or topical medication. Prospective participants were advised that the researchers were investigating medication compliance and the aids and barriers they may have experienced in administering medication to their dogs. During the informed consent process, clients were further informed that the study findings would be used to educate and assist pet owners in completing treatment instructions. Owners were also advised that they were free to exit the study at any time. Dogs prescribed medication by veterinary specialists at the practice, given their prescribed medication in-hospital by staff, or who died or were euthanized after consent and before completion of data collection were excluded.

### 2.3. Dissemination of Questionnaires

Following consent, clients were given a unique URL link to the online survey at the time of the initial visit and dispensing of prescribed medications and their hospital patient number to allow prescriptions to be matched to individual patients without identifying the owner to the investigators. Clients were asked to complete the survey after the prescription was provided. Participating owners were contacted by a hospital team member two weeks later, either in person at a recheck appointment or via telephone or email after the course of medication. The research team member in contact with the client was not involved in the blinded analyses of the results.

### 2.4. Data Analysis

Data from the online survey were imported as a spreadsheet CSV file into R statistical software (Version 3.6.1, R Development Core Team 2020, R Foundation for Statistical Computing, Vienna, Austria). Entries that failed the inclusion criteria and responses that were not sufficiently complete to evaluate outcomes were excluded from further analysis.

Descriptive statistics were summarized for all quantitative study variables. The distribution of continuous variables was evaluated for normality by the Shapiro–Wilk test. The mean and standard deviation were calculated for continuous data that were normally distributed. For data that were not normally distributed, median and interquartile range values were calculated. For the Likert scale questions, median and mode are reported.

Univariable logistic regression analyses were performed to assess factors associated with noncompliance and dog medication administration. The binary outcome measure, noncompliance (yes/no), was defined as failing to give the prescribed medication for the duration of the prescription or at the specified intervals or failing to administer the treatment. Noncompliance was identified by comparing the prescription directions in the veterinary record with the client’s recorded responses. Any deviation from the veterinarian’s instruction (i.e., dosage, frequency, or completion of the course of medication) within the prescription period was identified as noncompliance.

Explanatory variables encompassing client, animal, veterinary visit, and medication factors were explored for their associations with the outcome. An initial univariable logistic regression was performed for each potential explanatory variable, and *p*-values were calculated using the Wald test. Each predictor variable returning a *p*-value < 0.20 from the univariate modeling was considered for inclusion in a multivariable model. A stepwise backward elimination procedure was then performed whereby predictive values with the least significant *p*-value were successively removed until all variables in the final model had a Wald’s *p*-value < 0.05. Basic diagnostic statistics, including fitted and standardized residuals and leverage, were examined for adherence to model assumptions. No influential points were detected. The models were compared using the ANOVA of the deviance function in R, and the model with the lowest Akaike information criterion (AIC) was chosen [15]. The findings are presented as odds ratios (ORs) and confidence intervals (95% CIs) for each predictive variable.

## 3. Results

### 3.1. Survey Response

Owners of 151 dogs agreed to participate in the study.

### 3.2. Descriptive Data, Predictive and Outcome Variables

The breed category, sex, weight, and age of study dogs are summarized in Table 1. Patient breed, weight, and sex data were available for 117/151 dogs. As a result of sample size, dogs were grouped into three categories based on weight: large breed, medium breed, and small breed. A complete listing of dog breeds in this study can be found in the Supplementary Materials. The median weight of dogs in the study was 23.0 kg (IQR 22.0 kg; range 2.2 to 73 kg), and the median age was 6.6 years (IQR 7.8 years; range 2 to 16.5 years).

Table 2 summarizes the frequency of owners’ responses to the survey on medication administration to their dogs in New Zealand. The proportion of surveyed client dog owners who were noncompliant with medication instructions was 47% (71/151), resulting in an absolute compliance rate of 53% (80/150).

Respondents were most commonly female, aged between 51 and 60 years, New Zealanders of European ethnicity, and educated to a university level. Three-quarters of dog owners reported prior experience in the ownership of multiple pets of multiple species. Most (174/151, 90%) had prior experience managing pet illness, and a tenth reported they had training in animal health. Almost half the clients had prior experience with orally medicating pets, and most of these reported they had experience with administering medication both orally and topically. Most respondents administered the medication themselves. All respondents answered that they had a good understanding of the reason the medication was prescribed, with 95% (144/151) responding that they understood either “very well” or “extremely well”.

Most respondents strongly agreed that the veterinarian had spent enough time explaining the reason for the prescription and that the veterinarian had explained the reason for the medication well. Most clients were shown how to administer the medication by a veterinarian or veterinary student (53%; 79/151), although almost half reported that “nobody” showed them how to give the medication(s).

There were 69 different medications prescribed. Most of the dogs in this study were prescribed at least one oral medication. Medication classes prescribed (as reported by the client) are listed in Table 2. Fifty-five (36.4%) clients were prescribed more than one medication for their dog. The frequency of administration was twice a day for half the dogs. Less than a third of clients reported giving medication directly into their dog’s mouth, while most reported administering the medication with food, with a treat, or a combination of these methods.

Approximately a third of clients reported challenges giving the prescribed medication. The most common problem listed by respondents was “my pet was resistant to my efforts to medicate him/her”. Other barriers identified by respondents include treatment duration, inability to give medication with food, inconvenient dosing regimen, number of medications prescribed, and the complexity of the treatment regimen. Several owners reported diarrhea in their pets and associated this with the prescribed medication—which they then stopped. Multiple owners also reported altering the medication in some manner in order to administer it. This included opening capsules, crushing tablets, and altering recommended administration schedules. Owner-reported aids in medicating their dogs were grouped into the following categories: food, technique, behavioral modification, positive reinforcement, and multiple aids (Table 2).

Survey participants were asked what went well with medication administration and were encouraged to offer any “tricks” or techniques that they employed that made medicating their pet easier. These responses were collected via free-text entry with a total of 147 respondents. Several common themes emerged from these answers. The majority of these respondents (55.1%; 81/147) reported that food or a treat was helpful in assisting oral medication. These food-related responses ranged from putting the medication directly into the food to finding a particular food or treat to help deliver the medicine. Of the 26 respondents who described a particular technique as helpful, eight mentioned some form of restraint as a positive influence. Fifteen respondents (10.3%) reported an attribute of the medication itself as a positive feature. For example, several respondents noted the inclusion of a measured syringe for liquid suspensions as helpful. Of the respondents who reported that there were no tricks or techniques that improved the administration, free-text comments such as “I just put it in his mouth” and “it was straightforward” predominated. Some owners said their dog would “eat anything”. Others took a more direct approach, stating the process was straightforward or “I just gave it”. Several directly commented that the demonstration by the veterinary team was instructive enough and precluded the use of any aids, simply stating, “I did as shown”.

### 3.3. Univariate and Multivariable Logistic Regression

The results of the univariate logistic regression analyses are presented in Table 3. Univariate screening identified the following as inclusion variables for the multivariate model: breed category of dog, sex of dog, age of dog, client experience with medicating pets, who administered medication to the pet, prescription of oral antimicrobials, multiple medications prescribed, highest medication frequency as reported by the client, highest medication frequency as reported by the veterinarian, who showed the client how to give the medication, and owner-reported aids to medicating their pet (technique/training, restraint, nothing).

The results of the multivariable logistic regression analysis are reported in Table 4. Due to missingness and model errors, it was not feasible to include all variables identified in the multivariable analysis. Models for dog-associated factors and medication-associated factors were investigated separately. In the final multivariable model, the age of the dog and doing “nothing” as an aid to medication were associated with noncompliance.

## 4. Discussion

A significant proportion (47%) of surveyed client dog owners were noncompliant with medication instructions. Compliance in this study was defined as 100% or absolute and, using this as a basis for comparison to other studies reporting data in a similar manner, this proportion of noncompliance among dog-owning clients is lower than values reported in previous studies in dogs (56% to 89%) [4,6,13,16,17]. Other canine studies have reported compliance rates as median values, making a comparison of the current findings with those studies challenging [5,7]. The clientele of the MUVTH is overrepresented by individuals with animal experience (veterinary students, nurses, veterinary educators) and, therefore, compliance may be overestimated when compared to the clientele of other New Zealand private practice clinics.

This study aimed to identify factors associated with the noncompliance of owners with veterinary recommendations for medication administration to dogs by examining client factors, animal factors, veterinary visit factors, and medication factors. A significant association (*p* < 0.05) was found between the age of the dog and noncompliance, with increasing age having a protective effect on compliance, an animal factor that has not been previously reported to the authors’ knowledge. There could be several possible explanations for this finding. Firstly, like humans, older animals are more likely to have medical conditions that require medication and possibly more experienced in receiving treatment [18]. This could lead to a higher awareness and understanding among owners about the importance of medication compliance for their older pets. Additionally, the potential chronic nature of treatments in older animals and the standard of geriatric care for dogs may provide more opportunities for client interaction with the veterinary team [1]. The majority of dogs in this study (61%) were over five years old, with one-third over ten years old. During these veterinary interactions, verbal explanations of the medication effects and repeating important medication information to the client may contribute to improved compliance [6]. This suggests that treatment compliance in animals can be enhanced by scheduling regular follow-up visits, either virtually or in person, to ensure the correct implementation of the treatment by the owner. Adjusting the therapy based on the animal’s response and actively involving the owner in the treatment plan are also recommended strategies. Furthermore, older animals with acute or chronic conditions may pose a greater social risk to clients who are noncompliant [9]. Owners may feel a desire to showcase a pet in good health, and neglecting the care of a dependent animal could be viewed as worse than not taking care of oneself.

In human medicine, the relationship between age and compliance is complex and may be closely associated with the specific disease being treated. Most studies suggest that medication adherence increases with age in humans [19,20]. Older patients also tend to have greater illness severity, which can be predictive of compliance [21,22]. Similarly, in dogs, the specific disease being treated may influence owner compliance [4]. Diseases commonly associated with geriatric animals, such as canine osteoarthritis, may significantly impact a client’s daily routine and serve as a strong motivator for compliance [23].

A significant association (*p* < 0.05) was also found between noncompliance and the aids that clients used to assist in medication administration. Not employing different methods (i.e., “nothing”) as an aid to medication administration was negatively associated with noncompliance. Respondents who did not report using any aids to medicate their dog may simply have had a patient who was more accepting of medication.

Prior studies in dogs have demonstrated that time spent with the veterinarian and, specifically, repeating the most important instructions and verbal explanation of the effect of the medication improved compliance [4,6]. While most respondents in this study were satisfied with the time spent by the veterinarian and the degree to which the medication was explained, the depth of information delivered in these visits likely varied. The use of printed or video instructional tools for client education remains inconsistent within the MUVTH. Pet owners may have been unaware of the options or methods available to assist them in medicating their animals. The choice of “nothing” as an aid to medicating their pet may also reflect the owner’s general inability to do so. The most frequently reported barrier to medication administration in this study was a resistant pet. The owner may have employed multiple techniques only to find that none of the suggested methods were successful. Further investigation into this human factor is warranted to determine what, if any, options these pet owners would elect to employ if given the opportunity.

Many owners rely on food to administer medications to their pets, and over half of the clients in this study used food or a treat as an aid in administering their dog’s medication [23]. Emphasizing the popularity of this practice, instructions not to give medication with food resulted in a low compliance rate (22%) in one veterinary study [7]. Thus, the palatability (or another quality) of the medication in this study may have impacted the clients’ ability to administer it. As an example, 63% of dogs in this study were prescribed an anti-inflammatory/pain reliever. The nonsteroidal anti-inflammatory drugs prescribed in the MUVTH are provided as a flavored tablet or suspension, likely contributing to the ease of administration of that class of medication. This is supported by others who have found that highly palatable chewable drug formulations markedly increase canine medication compliance [24]. Free text answers from this cohort of owners who reported not using an aid in medicating their dogs may also provide some insight into this finding. The temperament of the dog was commonly mentioned as a positive attribute, describing the animal as “calm” or “relaxed”. Dogs are reportedly less discriminating than cats, and fear of injury from the animal is not a reported barrier to compliance in the dog as it is in the cat [25,26].

A concerning proportion (47%) of the dog owners in this study replied “nobody” when asked who showed them how to administer the prescribed medication. This compares with 45% in a recent survey of cat owners [10]. The effect of demonstrating medication administration to pets by the veterinary team on compliance has not previously been examined. Incorrect techniques when delivering oral medication may limit the appropriate dose the animal receives, decrease the bioavailability of the medication, or cause injury to the pet or owner [27]. Curiously, however, this finding was not associated with noncompliance in the current study. Pet owners have been shown to misremember this information, but a closer look at the free text from this question may be more informative [7]. Interestingly, most dog owners who reported that nobody showed them how to give the medication indicated they did not require instruction due to prior training or personal confidence in the technique. Thus, the question design may have contributed to misleading results as these clients opted out of the offered demonstration.

Prior studies in veterinary medicine have focused on the delivery of oral or written information on prescribed medicines and how to administer them, but no canine studies have modeled the technique for owners [5,6,7]. To improve client compliance with directions for medication administration to dogs, it is important to educate pet owners or caregivers about the importance of medication adherence and provide clear instructions on how to properly give the medication [6,23]. This may include demonstrating how to give the medication, explaining the proper dosage and frequency, explaining the benefits of therapy, and providing information on any potential side effects or interactions with other medications [28,29]. Veterinary clients may recall only a portion of the information provided on discharge [30]. To counter this, veterinarians can use various tools and strategies to promote client compliance with medication administration, such as sending reminders about when to administer the medication, providing written instructions or visual aids, and offering follow-up appointments to check on the dog’s progress and adjust the treatment plan if necessary [30,31]. The in-clinic demonstration of medication to pets may be a future area of investigation for improvement of noncompliance.

Dog owners in this study were also asked to report what went well with medication administration. A majority (55%) responded that adding it to food or a treat was a positive influence in medicating their dog. While this is a popular tactic and likely improves the success of administration, ensuring complete and timely delivery of the entire dosage may prove difficult. If the dog is anorectic or the dosing schedule does not align with mealtimes, then the owner may not be able to consistently medicate their pet. Some medications may not be labeled for use with food, interfering with drug absorption [7]. Six respondents altered the medication (crushed a tablet, for example) to mix with their dog’s food, a tactic that may be inappropriate.

Challenges in medicating their pet were reported by 31% (47/151) of dog owners, and the primary reason listed resistance of the dog to being medicated (76%; 36/47). Difficulty getting the pet to take the medication is a reported barrier to compliance in dogs [23]. It has been previously suggested that length of treatment is also a barrier to compliance, but treatment duration was an uncommon factor listed by owners in this study [21]. Busy lifestyles or convenience have also been suggested as barriers to compliance in the previous literature [16,23]. However, only 4% (2/47) of respondents associated that the convenience of the dosing regimen was a challenge for them in medicating their dog, and this was not significantly associated with noncompliance in this study.

The study design, a blinded client survey, presented several limitations. While self-reporting is an indirect measure of medication compliance, it has been shown to be as effective as other indirect measures, including pill counts and refill rates, and is the most practical approach for studies in veterinary medicine [32,33]. More precise estimates of medication adherence can be gained via direct methods (e.g., observation, drug or metabolite blood concentrations). However, such methods are expensive, burdensome or invasive, or not applicable to veterinary medicine [18,34]. Owner self-reporting in veterinary compliance studies may overestimate compliance compared to other measurement methods [7,17]. As enrolment in the study was voluntary, more compliant owners may have been more likely to be respondents. The reliance on a convenience sample of pets may have led to an underrepresentation of noncompliant owners. While the survey was offered within two weeks of the MUVTH visit, the retrospective nature of the survey may have contributed to pet owner recall bias for self-reported data. The lack of a more standardized patient call-back routine to remind clients about rechecks after prescriptions may have decreased involvement in the study. Clients not completing the entire survey at their discretion and question design not forcing answers led to missing data. Lastly, the hospital’s clientele is overrepresented with individuals with animal handling experience (veterinary students, nurses, veterinary educators) and, therefore, may have overestimated compliance when compared to the clientele of other practices.

## 5. Conclusions

This is the first study to associate age with compliance in dogs. With an overall compliance rate of 53%, the results of this study confirm the need for approaches that improve client compliance with veterinary instructions for the medication of their dogs. Our findings underscore the importance of providing standardized instruction and guidance for all dog-owning clients, including the demonstration of medication administration. Ensuring that oral medications prescribed for dogs are palatable and easy to administer before patient discharge may assist in improving compliance in this species.

## Figures and Tables

**Table 1:** Demographic data on dogs of respondents to a survey on compliance with medication administration in New Zealand. Data were available for 117/151 dogs.

Variable Name	Category	Count (%)
Breed grouping	Small breed dogs (<10 kg)	35 (30%)
	Medium breed dogs (>10 kg and <20 kg)	27 (23%)
	Large breed dogs (>20 kg)	55 (47%)
Sex of dog	Neutered males	43 (36.7%)
	Spayed females	45 (38.5%)
	Intact males	13 (11.1%)
	Intact females	16 (13.7%)

**Table 2:** Frequency table of responses to a survey on compliance with administration instructions for the medication to dogs in New Zealand. Data were available for 151 dogs.

Variable Name	Category	Count (%)
*Client factors*		
Prior experience with pet ownership	First dog	24 (16)
	Multiple dogs	17 (11)
	Multiple pets, multiple species	110 (73)
	Total	151 (100)
Prior experience with pet illness	This pet	28 (19)
	Other pets of the same species	28 (19)
	Multiple species	63 (42)
	Training in animal health	17 (11)
	No experience	15 (10)
	Total	151 (100)
Prior experience with medicating pets	No	4 (3)
	Yes, oral medication	72 (48)
	Yes, eye or ear medication	14 (9)
	Skin	5 (3)
	Yes, all routes	56 (37)
	Total	151 (100)
Client gender	Male	36 (24)
	Female	115 (76)
	Prefer not to answer	0 (0)
	Total	66 (100)
Client age group	<30 years old	23 (13)
	31–40 years old	18 (12)
	41–50 years old	40 (27)
	51–60 years old	46 (30)
	>60 years old	23 (15)
	Prefer not to answer	1 (0.7)
	Total	151 (100)
Client ethnic group	New Zealand European	112 (74)
	Other European	18 (12)
	Māori	11 (7)
	Asian	8 (5)
	Prefer not to answer	2 (1)
	Total	151 (100)
Client educational qualification (highest)	High school	35 (23)
	University	66 (44)
	Postgraduate	34 (23)
	Other	14 (9)
	Prefer not to answer	2 (1)
	Total	151 (100)
Client annual income	<NZD 14,000	11 (7)
	NZD 14,000–NZD 48,000	11 (7)
	NZD 48,000–NZD 70,000	19 (13)
	NZD 70,000–NZD 100,000	24 (16)
	>NZD 100,000	17 (11)
	Prefer not to answer	69 (46)
	Total	151 (100)
Client disability	No	149 (99)
	Yes	1 (1)
	Prefer not to answer	1 (1)
	Total	151 (100)
Who administered medication to the dog?	Myself	91 (60)
	Myself plus others	57 (38)
	Other	3 (2)
	Total	151 (100)
Client understanding of the reason the medication was prescribed	Extremely well	106 (70)
	Very well	38 (25)
	Moderately well	7 (5)
	Slightly well	0 (0)
	Not well at all	0 (0)
	Total	151 (100)
*Veterinary visit factors*		
The veterinarian spent enough time explaining	Strongly agree	131 (87)
	Somewhat agree	15 (10)
	Neither agree nor disagree	2 (1)
	Somewhat disagree	2 (1)
	Strongly disagree	0 (0)
	Total	150 (100)
	Missing	1
The veterinarian explained the reason for the medication well	Strongly agree	130 (89)
	Somewhat agree	18 (12)
	Neither agree nor disagree	1 (1)
	Somewhat disagree	1 (1)
	Strongly disagree	0 (0)
	Total	150 (100)
	Missing	1
Who showed how to give the medication(s)?	Veterinarian	61 (41)
	Veterinary Student	18 (12)
	Nobody	71 (47)
	Total	150 (100)
	Missing	1
*Medication factors*		
Medication class and route prescribed as reported by the client	Anti-inflammatory/pain relief (oral)	95 (63)
	Antimicrobial (oral)	38 (25)
	Behavioral (oral)	8 (5)
	Other oral medication	40 (27)
	Topical medication	32 (21)
	Multiple medications (oral and topical)	55 (36)
Frequency of medication	Once a day or every 24 h	43 (29)
	Twice a day or every 12 h	78 (53)
	Three times a day or every 8 h	12 (8)
	Other	15 (10)
	Total	148 (100)
	Missing	3
Length of prescription as reported by the client	Two days	4 (3)
	Three days	5 (3)
	Four days	5 (3)
	Five days	21 (14)
	Six days	0 (0)
	Seven days	27 (18)
	8 to 14 days	38 (25)
	15 to 21 days	6 (4)
	22 to 28 days	3 (2)
	42 to 364 days	13 (9)
	Ongoing	23 (15)
	Not specified	6 (4)
	Total	151 (100)
Oral medication prescribed	Yes	132 (88)
	No	19 (12)
	Total	151 (100)
How was oral medication administered	Directly into the dog’s mouth	40 (31)
	With regular dog food	30 (23)
	With a treat	39 (30)
	Combination of methods	20 (15)
	Other	2 (2)
	Total	131 (100)
	Missing	1
Challenges with oral medication	Yes	47 (31)
	No	103 (69)
	Total	150 (100)
	Missing	1
Missed doses of oral medication	Yes	39 (30)
	No	93 (70)
	Total	132 (100)
	Missing	19
Topical medication prescribed	Yes	45 (30)
	No	105 (70)
	Total	150 (100)
	Missing	1
Challenges with topical medication	Yes	12 (27)
	No	33 (73)
	Total	45 (100)
Missed dose of topical medication	Yes	19 (42)
	No	26 (58)
	Total	45 (100)
Medicating pet was a difficult experience for client or pet	Strongly Agree	8 (17)
	Somewhat Agree	19 (41)
	Neither agree nor disagree	8 (17)
	Somewhat disagree	7 (15)
	Strongly disagree	4 (9)
	Total	46 (100)
Owner-reported aids in medicating dogs	Food	81 (55)
	Technique	26 (18)
	Medication attribute	15 (10)
	Behavioral modification	14 (10)
	Multiple aids used	12 (8)
	No/Nothing	20 (14)
	Total	147

**Table 3:** Results of univariate logistic regression analyses of associations between noncompliance and dog factors and medication factors. Variables with a *p*-value < 0.25 were selected for initial inclusion in the multivariate logistic regression model based on the results of univariate logistic regression analyses of associations between noncompliance and animal, client, veterinary visit, and medication factors. Data were collected from an online survey on owner compliance with medication administration to dogs in New Zealand (*n* = 151) between 2019 and 2020.

Variable Name	Category	Est ^1^	SE ^2^	OR ^3^	95%CI ^4^	*p* ^5^
Breed category	Large	Ref				
	Medium	3.24		25.60	5.99–181.01	<0.001
	Small	3.16		23.50	5.90–158.95	<0.001
Sex of dog	Fem/spayed	Ref				
	Female	3.87	1.14	48.00	7.21–975.66	<0.001
	Male	3.94	1.17	51.20	7.32–1065.4	<0.001
	M/neutered	3.79	1.06	44.44	8.33–827.12	<0.001
Age of dog (years)		−0.10	0.05	0.90	0.82–0.99	0.03
Client experience administering medication	All routes	Ref				
	Eye/ear	−0.30	0.60	0.74	0.22–2.45	0.62
	None	0.80	1.19	2.23	0.27–46.60	0.50
	Oral	−0.85	0.37	0.43	0.21–0.88	0.02
	Skin	1.09	1.15	2.97	0.41–60.13	0.35
Who gave medication to pet	Myself	Ref				
	Other	0.27	1.43	1.31	0.05–33.76	0.85
	Myself and others	0.48	0.34	1.62	0.83–3.20	0.16
Medication class	No	Ref				
	Antimicrobial	0.60	0.39	1.82	0.86–3.93	0.12
Multiple medications	No	Ref				
	Yes	0.44	0.35	1.56	0.79–3.10	0.20
Highest med frequency per vet		0.30	0.25	1.36	0.83–2.27	0.22
Who explained medication	Veterinarian	Ref				
	Nobody	−0.60	0.35	0.55	0.27–1.10	0.09
	Student	0.28	0.55	1.33	0.46–4.05	0.61
Who explained: categorized	Veterinary team	Ref				
	Nobody	−0.42	0.34	0.66	0.34–1.27	0.22
Medication aid	No	Ref				
	Technique	0.83	0.46	2.29	0.95–5.82	0.07
Medication aid	No	Ref				
	Restraint	−0.95	0.70	0.39	0.08–1.41	0.18
Medication aid	No	Ref				
	“Nothing”	−1.42	0.67	0.24	0.05–0.80	0.03

^1^ Coefficient estimate; ^2^ standard error; ^3^ odds ratio; ^4^ 95% confidence interval; ^5^ *p*-value for variable.

**Table 4:** Results of multivariable logistic regression analyses of associations between noncompliance and dog factors and medication factors. Data were collected from an online survey on medication administration to dogs in New Zealand (n = 151) between 2019 and 2020.

Variable	Category	Est ^1^	SE ^2^	OR ^3^	95%CI ^4^	*p* ^5^
Dog age		−0.10	0.05	0.90	0.82–0.99	0.030
Medication aid	No	Ref				
	Nothing	−1.42	0.67	0.24	0.05–0.81	0.030

^1^ Coefficient estimate; ^2^ standard error; ^3^ odds ratio; ^4^ 95% confidence interval; ^5^ *p*-value for variable.

## Data Availability

Further inquiries can be directed to the corresponding author(s).

## Supplementary Materials

The following supporting information can be downloaded at https://www.mdpi.com/article/10.3390/ani14172557/s1, Supplementary File S1: Survey form completed by clients participating in the study of dog owner medication compliance.

## References

[1] Abood S.K. (2007). Increasing adherence in practice: Making your clients partners in care. Veter. Clin. N. Am. Small Anim. Pr..

[2] Ramió-Lluch L., Brazís P., Ferrer L., Puigdemont A. (2020). Allergen-specific immunotherapy in dogs with atopic dermatitis: Is owner compliance the main success-limiting factor?. Vet. Rec..

[3] Wareham K.J., Brennan M.L., Dean R.S. (2019). Systematic review of the factors affecting cat and dog owner compliance with pharmaceutical treatment recommendations. Vet. Rec..

[4] Grave K., Tanem H. (1999). Compliance with short-term oral antibacterial drug treatment in dogs. J. Small Anim. Pract..

[5] Amberg-Alraun A., Thiele S., Thiele S., Kietzmann M. (2004). Study of the pet-owners compliance in a small animal clinic. Kleintierpraxis.

[6] Verker M., van Stokrom M., Endenburg N. (2008). How can veterinarians optimise owner compliance with medication regimes. Eur. J. Companion Anim. Pract..

[7] Adams V.J., Campbell J.R., Waldner C.L., Dowling P.M., Shmon C.L. (2005). Evaluation of client compliance with short-term administration of antimicrobials to dogs. J. Am. Vet. Med. Assoc..

[8] Boda C., Liège P., Rème C.A. (2011). Evaluation of owner compliance with topical treatment of acute otitis externa in dogs: A comparative study of two auricular formulations. Int. J. Appl. Res. Vet. Med..

[9] Maille V., Hoffmann J. (2013). Compliance with veterinary prescriptions: The role of physical and social risk revisited. J. Bus. Res..

[10] Taylor S., Caney S., Bessant C., Gunn-Moore D. (2022). Online survey of owners’ experiences of medicating their cats at home. J. Feline Med. Surg..

[11] Salgado-Caxito M., Benavides J.A., Atero N., Córdova-Bürhle F., Ramos R., Fernandez M., Sapiente-Aguirre C., Mardones F.O. (2023). Preventive healthcare among dogs and cats in Chile is positively associated with emotional owner-companion animal bond and socioeconomic factors. Prev. Vet. Med..

[12] Negash R., Li E., Jacque N., Novicoff W., Evans S.J.M. (2024). Owner experience and veterinary involvement with unlicensed GS-441524 treatment of feline infectious peritonitis: A prospective cohort study. Front. Vet. Sci..

[13] Booth S., Meller S., Packer R.M., Farquhar R., Maddison J.E., Volk H.A. (2021). Owner compliance in canine epilepsy. Vet. Rec..

[14] Van Vlaenderen I., Nautrup B.P., Gasper S.M. (2011). Estimation of the clinical and economic consequences of non-compliance with antimicrobial treatment of canine skin infections. Prev. Vet. Med..

[15] Field A., Miles J., Field Z. (2012). Discovering Statistics Using R.

[16] Bomzon L. (1978). Short-term antimicrobial therapy—A pilot compliance study using ampicillin in dogs. J. Small Anim. Pract..

[17] Barter L.S., Watson A.D., Maddison J.E. (1996). Owner compliance with short term antimicrobial medication in dogs. Aust. Vet. J..

[18] Epstein M., Kuehn N.F., Landsberg G., Lascelles B.D.X., Marks S.L., Schaedler J.M., Tuzio H., Senior Care Guidelines Task Force (2005). AAHA senior care guidelines for dogs and cats. J. Am. Anim. Hosp. Assoc..

[19] Park D.C., Hertzog C., Leventhal H., Morrell R.W., Leventhal E., Birchmore D., Martin M., Bennett J. (1999). Medication adherence in rheumatoid arthritis patients: Older is wiser. J. Am. Geriatr. Soc..

[20] Krueger K., Botermann L., Schorr S.G., Griese-Mammen N., Laufs U., Schulz M. (2015). Age-related medication adherence in patients with chronic heart failure: A systematic literature review. Int. J. Cardiol..

[21] Kim S.J., Kwon O.D., Han E.B., Lee C.M., Oh S.-W., Joh H.-K., Choi H.C. (2019). Impact of number of medications and age on adherence to antihypertensive medications: A nationwide population-based study. Medicine.

[22] DiMatteo M.R., Haskard K.B., Williams S.L. (2007). Health beliefs, disease severity, and patient adherence: A metaanalysis. Med. Care.

[23] Maddison J.E. (2011). Medication compliance in small animal practice (2022). Vet. Irel. J..

[24] Visser M., Walsh K., King V., Caneva L. (2022). Acceptance of oclacitinib maleate (Apoquel^®^) chewable tablets in client-owned dogs with allergic and atopic dermatitis. BMC Vet. Res..

[25] Thombre A.G. (2004). Oral delivery of medications to companion animals: Palatability considerations. Adv. Drug Deliv. Rev..

[26] Murphy L.A., Wang M.L., O’Malley B., Schrope D.P., Allen J.W., Chapel E.H., Nakamura R.K. (2022). A multi-center prospective evaluation of owner medication adherence for feline cardiovascular disease in the referral setting. J. Vet. Cardiol..

[27] Chapman E. (2018). The importance of client compliance and the influences upon client compliance when orally medicating cats. Vet. Nurs. J..

[28] Beco L., Guaguere E., Mendez C.L., Noli C., Nuttall T., Vroom M. (2013). Suggested guidelines for using systemic antimicrobials in bacterial skin infections (2): Antimicrobial choice, treatment regimens and compliance. Vet. Rec..

[29] Talamonti Z., Cassis C., Brambilla P.G., Scarpa P., Stefanello D., Cannas S., Palestrini C. (2015). Preliminary Study of Pet Owner Adherence in Behaviour, Cardiology, Urology, and Oncology Fields. Vet. Med. Int..

[30] Flegel T., Dobersek K., Bayer S., Becker L.F., Loderstedt S., Böttcher I.C., Dietzel J., Tastensen C., Kalliwoda T., Harkenthal M.A. (2024). Client’s understanding of instructions for small animals in a veterinary neurological referral center. J. Vet. Intern. Med..

[31] Barragry T. (2000). Prescription compliance: A veterinary perspective. Ir. Vet. J..

[32] Khdour M.R., Hawwa A.F., Kidney J.C., Smyth B.M., McElnay J.C. (2012). Potential risk factors for medication non-adherence in patients with chronic obstructive pulmonary disease (COPD). Eur. J. Clin. Pharmacol..

[33] Barter L.S., Maddison J.E., Watson A.D. (1996). Comparison of methods to assess dog owners therapeutic compliance. Aust. Vet. J..

[34] Osterberg L., Blaschke T. (2005). Adherence to medication. N. Engl. J. Med..

