# Supplementary material for: Factors Associated with Medication Noncompliance in Dogs in New Zealand
Source: Animals (Basel). 2024 Sep 3;14(17):2557. doi: 10.3390/ani14172557 (PMC11394019; doi:10.3390/ani14172557)
Supplement: Supplementary file 1 [file animals-14-02557-s001.zip › animals-3159196-supplementary.pdf]

Supplementary File S1: Survey form completed by clients participating in the study of dog owner medication compliance.

Start of Block: Demographic questions

Q1 What is your gender?

o Male (1)

o Female (2)

o Prefer not to answer (3)

Q2 What age group do you fit into?

o 18-20 years old (1)

o 21-30 years old (2)

o 31-40 years old (3)

o 41-50 years old (4)

o 51-60 years old (5)

o 61-70 years old (6)

o 71 years or older (7)

Q3 Which ethnic groups do you belong to? (check all that apply)

▢ New Zealand European (1)

▢ Other European (2)

▢ Maori (3)

▢ Cook Island Maori (4)

▢ Samoan (5)

▢ Tongan (6)

▢ Niuean (7)

▢ Chinese (8)

▢ Indian (9)

▢ Other (please specify) (10) ____

Q4 Which statement best describes your total annual income? (Check all that apply)

▢ Zero income (1)

▢ Up to $14,000 (2)

▢ $14,001 to $48,000 (3)

▢ $48,001 to $70,000 (4)

▢ $70,000 and over (5)

▢ Received pension (NZ superannuation, or equivalent overseas pension) (6)

▢ Received work and income benefit (either job seeker support, sole parent support, or supported living payment) (7)

Q5 What is your highest qualification?

o none (1)

o High School qualification (such as NCEA levels 1-3, school certificate, sixth form certificate, higher leaving certificate, university entrance) (2)

o University qualification (degree, diploma, certificate) (3)

o Postgraduate degree (4)

o Trade certificate (5)

o other (please specify) (6) ____

Q6 What is your prior pet ownership experience?

o This is my first pet (1)

o I have owned one pet before, of the same species (2)

o I have owned multiple pets before, but only of this species (cats ONLY, for example) (3)

o I have owned multiple pets of multiple species before (dogs AND cats, for example) (4)

Q7 What is your prior experience with pet illness? (Check all that apply)

▢ I've had to manage illness with this pet before (1)

▢ I've had to manage illness with other pets of this species before (2)

▢ I've had to manage illness in pets of multiple species before (3)

▢ I have training in animal health (please specify) (4)

▢ I have no prior experience in managing illness in pets (5)

Q8 One of the purposes of this study is to identify any potential barriers to medicating pets, and to help overcome those barriers. Do you have any physical disability or impairment that may hinder your ability to medicate your pet?

o yes (1)

o no (2)

o prefer not to answer (3)

Start of Block: Medication Questions

Q9 Which member of your family/household gave MOST of the medication to your pet?

o Myself (1)

- o Myself and/or one other person (2)
- o Myself and/or two other persons (3)
- o Myself and/or three other persons (4)
- o Other (please specify) (5) ____

Q10 How well do you understand the reason(s) why your veterinarian recommended the prescribed medication?

- o Extremely well (1)
- o Very well (2)
- o Moderately well (3)
- o Slightly well (4)
- o Not well at all (5)

Q11 Can you recall the name of the disease or condition that your pet was diagnosed with?

____

Q12 Most of the time, how often did you give your pet the prescribed medication?

- o Once a day or every 24 hours (1)
- o Twice a day or every 12 hours (2)
- o Three times a day or every 8 hours (3)
- o Other (please specify) (4) ____

Q13 I gave the prescribed medication to my pet for _________ days/weeks:

- o how many days (1) ____
- o how many weeks (2) ____

Q14 Did you miss any doses of the prescribed medication?

- o Missed most doses (1)
- o Missed frequent doses (2)
- o Missed some doses (3)
- o Missed hardly any doses (4)
- o Did not miss any doses (5)

Q15 I feel like the veterinarian spent enough time explaining the medication to me.

- o Strongly agree (1)
- o Somewhat agree (2)
- o Neither agree nor disagree (3)
- o Somewhat disagree (4)

- Strongly disagree (5)

Q16 I feel like the veterinarian explained the reason for the medication well.

- Strongly agree (1)
- Somewhat agree (2)
- Neither agree nor disagree (3)
- Somewhat disagree (4)
- Strongly disagree (5)

Q17 Who showed you how to give your pet their medication?

- Veterinarian (1)
- Veterinary Student (2)
- Other Staff member (3)
- Nobody (4)
- other (please specify) (5) ____

Q18 Were there any challenges giving the prescribed medication to your pet?

- yes (1)
- no (2)

Skip To: Q21 If Were there any challenges giving the prescribed medication to your pet? = no

Q19 Listed below are some common reasons why medication administration to pets is challenging. Did any of these apply to you? (Check all that apply):

- ▢ Method of administration was difficult (1)
- ▢ My pet was resistant to my efforts to medicate him/her (2)
- ▢ Label instructions were unclear (3)
- ▢ Treatment duration (4)
- ▢ I did not see a benefit to the medication (5)
- ▢ Complexity of treatment was difficult for me (6)
- ▢ Number of different medications prescribed (7)
- ▢ Medication was physically difficult for me to administer (8)
- ▢ Instructions from veterinary team were unclear (9)
- ▢ Unable to give medication with food (10)
- ▢ Dosage frequency was inconvenient for my lifestyle (11)
- ▢ Other (please specify) (12) ____

Q20 Medicating my pet was a difficult experience for myself and/or my pet.

- Strongly agree (1)
- Somewhat agree (2)
- Neither agree nor disagree (3)
- Somewhat disagree (4)
- Strongly disagree (5)

Q21 We are also interested in what went well with medication administration. Were there any tricks or techniques that you employed that made medicating your pet easier?

____

Q22 If you were prescribed oral medication for your pet, how did you give it most of the time? (Pick one)

- With my pets regular food (1)
- With a snack or a 'treat' (2)
- Directly into my pets mouth (3)
- A combination of these methods (4)
- An oral medication was not prescribed (5)
- Did not end up giving any (6)
- Other (please specify) (7) ____

Q23 Were you prescribed a topical medication?

- Yes (1)
- No (2)

Skip To: Q25 If Were you prescribed a topical medication? = No

Q24 If you were prescribed a topical medication, did you encounter any challenges administering it?

- Yes (please clarify) (1) ____
- No (2)

Q25 Please make any additional comments that you may have on the questionnaire, this study, or specific questions in the space provided below. Your input is welcomed.

____

End of Block: Medication Questions
